# The impact of balanced reciprocal translocation - 46,XX,t(7;17) (p13;q24) probably involving the SOX9 gene in the *in vitro* fertilization with own oocytes evaluated by preimplantation genetic testing or donated oocytes

**DOI:** 10.5935/1518-0557.20180066

**Published:** 2019

**Authors:** Pedro F.M. Peregrino, Alecsandra Gomes, Mariana Fujii, Tatiana C.S. Bonetti, Marcia Riboldi, Pedro A.A. Monteleone

**Affiliations:** 1 Monteleone - Centro de Reprodução Humana. São Paulo, Brasil; 2 Centro de Reprodução Humana Governador Mario Covas, Disciplina de Ginecologia, Departamento de Obstetrícia e Ginecologia, Hospital das Clinicas, Faculdade de Medicina, Universidade de São Paulo (FMUSP). São Paulo, Brasil; 3 Departamento de Ginecologia, Universidade Federal de São Paulo - Escola Paulista de Medicina (UNIFESP-EPM). São Paulo, Brasil; 4 Igenomix Brasil, Laboratório de Medicina Genética Ltda. São Paulo, Brasil

**Keywords:** preimplantation genetic testing (PGT), balanced translocations, *in vitro* fertilization (IVF)

## Abstract

Preimplantation genetic testing (PGT) for *in vitro* fertilization (IVF) - also known as PGT for Structural Rearrangements (PGT-SR) - has emerged as an option for at-risk couples carrying balanced translocations. The female in the couple featured in this case report is a carrier of a balanced reciprocal translocation who underwent IVF. PGT showed all her embryos were aneuploid. She subsequently had two cycles using donor oocytes, which ended in miscarriages.

## INTRODUCTION

Preimplantation genetic testing (PGT) with *in vitro* fertilization (IVF) has emerged as an important option for at-risk couples wishing to conceive healthy children without fatal or severely debilitating inherited disorders. The incidence of balanced reciprocal translocations ranges between 0.1-0.2%. Nonetheless, in most cases these derivative chromosomes do not lead to any significant loss of material, and therefore the vast majority of carriers do not exhibit abnormal phenotypes (Scriven *et al*., 1998).

However, balanced reciprocal translocations in germline cells may result in a variety of unbalanced translocations during the process of meiosis (Munné, 2005); thus, recurrent miscarriage is a common reproductive outcome for couples carrying a translocation due to aneuploid embryos (Suzumori & Sugiura-Ogasawara, 2010). PGT is a diagnostic option for couples carrying translocations, since it identifies euploid embryos prior to transfer, thus allowing the development of healthy babies (Munné *et al*., 2000).

The female in the couple featured in this case report is a carrier of a balanced reciprocal translocation submitted to IVF. PGT showed all her embryos were aneuploid. She subsequently had two cycles using donor oocytes, which ended in miscarriages.

## CASE DESCRIPTION

A 12-year-old female patient was referred to the Genetic Counseling Service of the University of São Paulo on account of short stature (136.5 cm, 3rd percentile), delayed bone age and skeletal anomalies including hypoplastic scapulae, thoracolumbar scoliosis, 11 pairs of ribs with hypoplasia of the first four pairs. Her intellectual development was normal. Chromosome analysis after G-banding revealed a balanced reciprocal translocation between the short arm of chromosome 7 and the long arm of chromosome 17, 46,XX,t(7;17) (p13;q24). At 31 years of age, her height (159 cm) and weight (54 kg) were around the 25^th^ centile and she returned for genetic counseling to assess the risk of having affected offspring (Fonseca *et al*., 2013). At 37 years of age, the patient was referred to our clinic, the Monteleone Center for Human Reproduction, São Paulo - Brazil, wishing to undergo *in vitro* fertilization with PGT to avoid the risk of having affected children.

**Figure 1:**
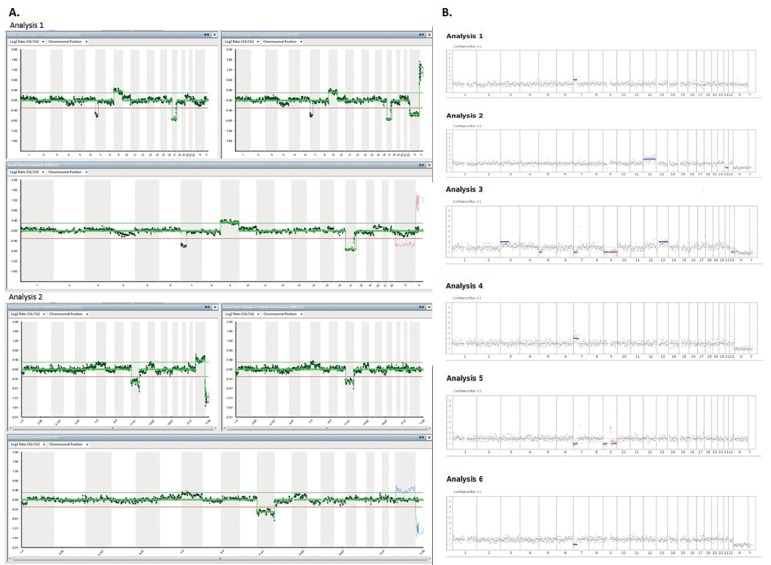
A. Images by CGHa of two embryos analyzed in the first IVF cycle; B. Images by NGS of six embryos analyzed in the second IVF cycle. CGHa: Comparative genome hybridization array. NGS: Next Generation Sequencing.

The patient underwent IVF + PGT-SR after signing an informed consent term. She underwent the first IVF cycle in May 2016. The patient was given recombinant FSH for ovarian stimulation; GnRH antagonist for pituitary blockage; and final oocyte maturation was triggered with recombinant hCG. Fourteen oocytes were harvested, 12 of which were mature (MII) and two at the germinal vesicle stage (GV). All MII oocytes were fertilized by ICSI using ejaculated sperm from her partner and cultured in standard conditions. Two embryos reached the blastocyst stage and were biopsied on day 5 of development for PGT-SR analysis. PGT was carried out at a reference laboratory by comparative genomic hybridization array (CGHa) for 24-chromosome analysis (Igenomix, Brazil) using standardized procedures. The results of blastocyst genetic analysis revealed anomalies: blastocyst 1 was a male presenting an unbalanced translocation and whole chromosome aneuploidies (-7p, +9, -17, XY) and blastocyst 2 was a female presenting only whole chromosome aneuploidies (+7, -11, XX) (Table 1 and Figure 1A).

**Table 1:** Results of embryo PGT.

IVF Cycle 1	Type of cell analyzed	PGT technique	Result
Blastocyst 1	Trophectoderm	CGHa	-7p, +9, -17, XY
Blastocyst 2	Trophectoderm	CGHa	+7, -11, XX
IVF Cycle 2			
Blastocyst 1	Trophectoderm	NGS	+7p, XX
Blastocyst 2	Trophectoderm	NGS	+12, -21, XY
Blastocyst 3	Trophectoderm	NGS	-7, -9, +13, -22, XY
Blastocyst 4	Trophectoderm	NGS	+7p, XY
Blastocyst 5	Trophectoderm	NGS	-7p, -9, XX
Blastocyst 6	Trophectoderm	NGS	-7p, XY

CGHa Comparative genome hybridization array. NGS: Next Generation Sequencing

The patient underwent a second ovarian stimulation cycle in July 2016, using the same protocol as before and 13 oocytes were collected: 11 MII, 1 MI and 1 degenerated oocyte. Twelve oocytes were fertilized by ICSI and six blastocysts were biopsied on day 5 of development. PGT-SR analysis was carried out in the same reference laboratory by Next Generation Sequencing (NGS) for 24-chromosome analysis (Igenomix, Brazil). All embryos presented abnormalities (Table 1 and Figure 1B) and none was transferred. The patient was advised to start a new IVF cycle using donated oocytes.

**Figure 2:**
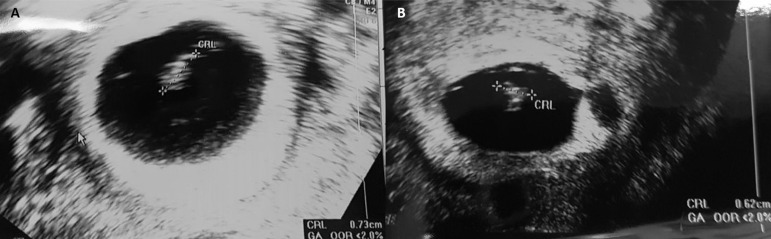
A.Ultrasound image of a gestational sac in the first IVF cycle with oocyte donation; B. Ultrasound image of a gestational sac in the second frozen-thawed embryo transfer with oocyte donation.

In March 2017, the couple decided to undergo a cycle using donor oocytes on a shared oocyte donation protocol. The patient received eight donor oocytes after endometrial preparation with estradiol and progesterone. Seven oocytes fertilized by ICSI with partner sperm developed until day 5. Two blastocysts were transferred and five were cryopreserved. bHCG levels measured 9 and 11 days after transfer were 76 IU and 204 IU, respectively. Ultrasound examination four weeks after embryo transfer showed a gestational sac (Figure 2A). Ultrasound examination eight weeks after embryo transfer showed a gestational sac without fetal heartbeat. 

In October 2017, the patient underwent a frozen-thawed embryo transfer with embryos cryopreserved in the previous oocyte donation cycle. After endometrial preparation, she had one top-quality blastocyst (grade 6) transferred. Eight days after the transfer the patient had a bHCG level of 250 IU. Ultrasound examination six weeks after the transfer procedure revealed a gestational sac with fetal heartbeat (Figure 2B). Ultrasound examination eight weeks after embryo transfer showed a gestational sac without fetal heartbeat. 

## DISCUSSION

The case described herein showcased the application of PGT-SR to identify normal embryos for transfer in a patient presenting a balanced reciprocal translocation between the short arm of chromosome 7 and the long arm of chromosome 17, 46,XX, t(7;17) (p13;q24). All embryos analyzed in two ovarian stimulation cycles had abnormal genetic profiles and most of them (7/8, 87.5%) had alterations in chromosome 7 associated or not with other aneuploidies. Another study applied NGS technology to identify chromosomally normal embryos for transfer in PGT for patients with translocations. In eight of the 21 patients, all embryos were abnormal as a result of aneuploidies, unbalanced translocations or both, indicating that patients with chromosome translocations are at high risk of producing unbalanced embryos with higher risk of incidental aneuploidies (Zhang *et al*., 2016).

PGT-SR remains challenging. A previous study evaluating couples with a history of recurrent pregnancy loss associated to reciprocal translocations demonstrated that individuals submitted to PGT and IVF had the same accumulated live birth rates of couples who conceived naturally (Ikuma *et al*., 2015). Also, when performing PGT, embryos may be abnormal due to unbalanced segregation of the two involved chromosomes; additionally, embryos diagnosed as balanced still face the possibility of additional incidental aneuploidies in other chromosomes that are known to commonly arise from non-disjunction errors (Vanneste *et al*., 2009). In the case described here, the translocation presented by the patient involved chromosomes 7 and 17. Seven of the eight embryos produced had alterations involving chromosome 7 associated or not with other chromosome aneuploidy, and only one embryo was aneuploid with no involvement of chromosomes 7 or 17. None of their own embryos was transferred.

Our patient received two other embryo transfers from donated oocytes, thus mitigating the risk of embryo aneuploidy and of carrying forward the maternal genetic disorder. However, both transfers resulted in miscarriages after eight weeks. The translocation breakpoint in chromosome 17 corresponded to the region of gene SOX9 mapped at 17q24.3, responsible for encoding a transcription factor with a role in chondrogenesis (Akiyama, 2008). Translocations involving this region cause a clinical condition known as campomelic dysplasia by presumptively altering SOX9 expression associated with skeletal defects (Mansour *et al*., 1995).

Despite the lack of reports of this specific reciprocal translocation associated with reproductive outcomes of carriers other than embryo chromosome unbalances, the case described herein prompted us to speculate that the occurrence of miscarriage might be associated to the translocation presented by the mother and not only by embryo genetic abnormalities.
